# Effects of High-Temperature-Pressure Polymerized Resin-Infiltrated Ceramic Networks on Oral Stem Cells

**DOI:** 10.1371/journal.pone.0155450

**Published:** 2016-05-19

**Authors:** Mathilde Tassin, Eric Bonte, Ludwig S. Loison-Robert, Ali Nassif, Tsouria Berbar, Stéphane Le Goff, Ariane Berdal, Michael Sadoun, Benjamin P. J. Fournier

**Affiliations:** 1 Faculty of Dentistry, Paris Descartes University, Sorbonne Paris Cité, France; 2 Laboratory of Molecular Oral Pathophysiology, INSERM UMRS 1138, Cordeliers Research Center, 15 rue de l'école de médecine, Paris, F-75006, France; Paris-Descartes; Pierre and Marie Curie; Paris-Diderot Universities, Paris, F-75006, France; 3 URB2i, Faculty of Dentistry, Paris Descartes University, Sorbonne Paris Cité, France; 4 Bretonneau Hospital, HUPNVS, AP-HP, Paris, France; 5 Faculty of Dentistry, Paris Diderot University, Sorbonne Paris Cité, France; Second University of Naples, ITALY

## Abstract

**Objectives:**

The development of CAD—CAM techniques called for new materials suited to this technique and offering a safe and sustainable clinical implementation. The infiltration of resin in a ceramic network under high pressure and high temperature defines a new class of hybrid materials, namely polymer infiltrated ceramics network (PICN), for this purpose which requires to be evaluated biologically. We used oral stem cells (gingival and pulpal) as an *in vitro* experimental model.

**Methods:**

Four biomaterials were grinded, immersed in a culture medium and deposed on stem cells from dental pulp (DPSC) and gingiva (GSC): Enamic (VITA®), Experimental Hybrid Material (EHM), EHM with initiator (EHMi) and polymerized Z100™ composite material (3M®). After 7 days of incubation; viability, apoptosis, proliferation, cytoskeleton, inflammatory response and morphology were evaluated *in vitro*.

**Results:**

Proliferation was insignificantly delayed by all the tested materials. Significant cytotoxicity was observed in presence of resin based composites (MTT assay), however no detectable apoptosis and some dead cells were detected like in PICN materials. Cell morphology, major cytoskeleton and extracellular matrix components were not altered. An intimate contact appeared between the materials and cells.

**Clinical Significance:**

The three new tested biomaterials did not exhibit adverse effects on oral stem cells in our experimental conditions and may be an interesting alternative to ceramics or composite based CAD—CAM blocks.

## Introduction

Loss of dental mineralized tissues caused by decay, erosion, congenital dental defects or trauma needs to be filled by biocompatible, functional, sustainable and if possible, aesthetic materials. Nowadays, Computer-Aided Design and Computer-Aided Manufacturing (CAD-CAM) devices are changing the actual pattern of restorative dentistry. This chairside method allows the practitioner to treat large dental defects in only one session while it needed with conventional means several appointments and the intervention of a dental technician. CAD-CAM method allies an optical impression with a direct processing of material block by a milling unit. CAD-CAM processes may use either composite or ceramics materials. The properties of CAD-CAM materials *per se* should be considered and optimized.

In this respect, ceramics present excellent biocompatibility, the best optical and good mechanical properties as well as sustainability [1]. The main pitfall of ceramics is the requirement and the difficulty of a re-intervention on altered fillings. Indeed, repair and rebonding may be difficult according to ceramics type. In CAD-CAM processing, another disadvantage of ceramics is their toughness which increases block milling time and the frequency of cutter replacement.

In contrast, resin-based composite blocks advantages are their easiness of both bonding and repair, with however optical and mechanical inferior properties compared to ceramics. Resin-based composites are composed of an organic polymer matrix with inorganic filler particles (usually glass). Their Young's modulus is close to that of dentin. The strengths and weaknesses of the materials are linked to their polymer organic components (Bisphenol A glycidylmethacrylate (Bis-GMA), triethylene glycol dimethacrylate (TEGMA), urethane dimethacrylate (UDMA) for example) [2]. These may constitute a biological risk in case of poor polymerization, with a release of monomers or their microleakage at the tooth-filling interface [3].

In order to circumvent material weakness, new materials have been designed few years ago. These are hybrids of the two formers [4]. Sintered ceramics porous blocks are silanated. Infiltration of the monomers through the network is performed under high pressure and high temperature, with the polymerization occurring at the same time These materials present better fracture toughness and flexural strength than classical composites [5]. This is partly due to their higher volume fraction filler (Table 1). Furthermore, high pressure–high temperature polymerization allows a better conversion rate, which limits putative monomer release in saliva [6]. Additionally, considering experimental evidences on Bisphenol A monomer toxicity in several pathologies [7], the present materials replaced bisphenol A based resins by bisphenol A-free resins (UDMA in this study).

**Table 1:** Composition and properties of the biomaterials.

NAME	MATRIX	FILLER	Vf (%)	CONFIGURATION	INITIATOR	POLYMERIZATION	Flexural strength (MPa)	Fracture toughness (Mpa.m1/2)
**Enamic (Vita)**	UDMATEGMA	Feldspathceramic enriched with aluminum oxide	75	Sintered block	Benzoyle Peroxyde	As-received CAD/ CAM blocks	150	1.5
**EHM**	100% UDMA	VITA Mark II	73.8	Sintered block, 800°C	None	HT/HP	288.3+/- 39.6	2.8+/- 0.7
**EHMi**	100% UDMA	VITA Mark II	73.8	Sintered block, 800°C	Di-tert-amyl peroxide	HT/HP	305.2+/- 53.7	2.7+/- 0.5
**Z100 (3M)**	Bis GMATEGDMA	Zirconia Silica0.6 μm	64.2	Mixing with matrix	Ternary initiator system	As-received CAD/ CAM blocks	138.2+/-24.3	0.8+/- 0.2

These alternative hybrid materials which combine the advantages of the former two materials are a newly explored research area. Founding studies on their mechanical properties placed them between classical ceramics and composite materials [8]. As the resin cannot be only infiltrated by capillary action, pressure has to be applied to improve the infiltration and to limit polymerization associated shrinkage. Higher infiltration pressure gave better mechanical properties [4], reduced the shrinkage by reducing free volume and may also limit the development of internal stress [5]. Although their components have already been independently evaluated in terms of cytotoxicity and biocompatibility, the biological safety of their association within the same material also needs to be evaluated here.

Material biocompatibility [9] may be first evaluated by general *in vitro* cytotoxicity testing[10]. Among cells used for these investigations, NHK or 3T3 fibroblasts are probably the most frequently used cells [10]. In this work, *in vitro* assays were performed in models which mimic *in vivo* interactions of oral cells with dental biomaterials. In this respect, oral mesenchymal stem cells have been isolated several years ago. These-ones are nowadays well characterized and identified as defined in 2006 [11]. Their advantages are an easy access, high proliferation rate combined with their ability to maintain a stable phenotype [12] [13]. Dental pulp (DPSC) and gingival stem cells (GSC) were selected here to evaluate the *in vitro* impact of biomaterials on cells exposed indirectly in the buccal and dental microenvironment. This study combined the characterization of their viability, proliferation, morphology, extracellular matrix production, inflammation and analyzed cell contacts with the biomaterial.

This experimental work focused on three (two of them are experimental, although already described Nguyen et al. [5]) High-Temperature-Pressure Polymerized resin-infiltrated glass-ceramic networks (RIGCN), using a resin-based composite (3M Co., St. Paul, MN, USA) as a reference. The hypotheses tested were that: 1- RIGCN are as biocompatible as classic dental composites and 2- they do not alter the phenotype of mesenchymal cells which are in direct or essentially indirect contact with materials within the dental and oral microenvironment.

## Materials & Methods

### Cell isolation

Human stem cells were extracted from dental pulp and gingiva as already described [14,15]. This research followed the statements found in the “Helsinki Declaration”, written informed consents were obtained. This was approved by the Office of Research Ethics of the University of British Colombia and by Paris local ethics committee.

### Cell culture

After isolation, cells were seeded in “proliferating medium” composed with classical Low Glucose Dulbecco’s modified eagle medium (DMEM 1X)—GlutaMAX^TM^(Gibco®, Invitrogen, Carlsbad, CA, USA) containing 10% Fetal Bovine Serum (FBS), 1% non-essential amino acids MEM 100X (Gibco®), 1% Penicillin-Streptomycin (10,000 U/mL) (Gibco®) and 0,5% Fungizone® Antimycotic (Gibco®) supplemented with 50 μg/mL 2-Phospho-L-ascorbic acid trisodium salt (Sigma-Aldrich, St. Louis, MO, USA) at 37°C in 5% CO_2_ incubator. To generate colony forming unit cultures (CFU) cells were seeded at low density (<100 cells/cm^2^) on 10 cm Petri dishes and cultured as above until colonies appeared.

### Colony efficiency assays

After 14 days of culture, cells were fixed using Phosphate Buffered Saline 1X (PBS; pH 7.4) containing 4% paraformaldehyde (PFA) / 5% sucrose for 10 min at 4°C; and then stained with 0.05% (w/v) crystal violet. Colonies were observed under microscope (EVOS Invitrogen).

### FACS analysis

Cells were detached using 0.02% Trypsin/EDTA solution in PBS and collected (5 min at 400 r.p.m.), washed in 3% FBS in PBS at 4°C, then incubated in solutions of CD29, CD31, CD45, CD90, CD105, CD146 and Stro1 markers in 3% BSA in PBS for 60 min at room temperature. Cells were then washed 3-times by centrifugation at 400 g for 5 min and resuspend them in 3% FBS/PBS. Cells were suspended again for observation.

### Osteogenic and adipogenic differentiation

Osteogenic and adipogenic differentiation were induced with selective media as already described [14]. Briefly, osteogenic differentiation was obtained after 14 days of culture in medium composed of “proliferating medium” supplemented with 10% Fetal Bovine Serum (FBS),50μg/mL 2-Phospho-L-ascorbic acid trisodium salt, 10 nM dexamethasone and 10 mM beta-glycerophosphate. Adipogenic differentiation was obtained after 14 days in medium composed of “proliferating medium” supplemented with 10μg/mLInsulin, 0,5 mM IBMX and 50 μM indomethacin and 1μM dexamethasone. Staining with Alizarin Red S and Oil Red O were used to assess osteogenic and adipogenic differentiation, respectively.

### Tested Biomaterials

Three RIGCN and one commercialized photopolymerized composite resin were tested. Chemical components and main physical properties of these biomaterials are summarized in Table 1. Briefly, the biomaterials were transformed in powder by grinding under cold conditions (-20°C) and sterilized by dry heat.

### Preparation of Biomaterial solutions

#### Basal medium without phenol red

The basal medium for biomaterials was the classical medium without phenol red and supplemented with 50 **μ**g/mL 2-Phospho-L-ascorbic acid trisodium salt (Sigma-Aldrich).

#### Biomaterial stock solutions (BSS)

After a titration test, a final concentration of 2 mg/mL was used for each biomaterial (Data not shown). Thus, the powder was added to the basal medium to prepare solutions of 10 mg/mL for each biomaterial and vortexed. The non-solubility property of the biomaterials in the basal medium was corrected by using 10% Ethanol in the stock solutions. This stock solution was added with a five times dilution to the cells.

#### 2% Agarose, low gelling temperature solution (ALGS)

Sterile Agarose low gelling temperature powder (Sigma-Aldrich) was added to the basal medium at 20 mg/mL. This solution was heat at 60°C for 5 min.

### Cell proliferation and viability assays

30.10^3^ cells were adhered for 2 h in 800μL of basal solution per well in a 24-well plates. Then, 200μL of BSS was added. Basal medium supplemented with 2% Ethanol served as control. The rate of proliferation was estimated by performing MTT analysis on day 0, 8, 14 and 21 as indicated by the supplier (Sigma Aldrich). Cell number was determined by measuring the absorbance (570 nm) on a multi-plate reader (TrisStar LB 941). Cell proliferation was also detected with Ki67 immunostaining (see below). Ki-67 indexes were assessed by determining the total number of Ki-67 immunofluorescent cells divided by the total number of DAPI stained nuclei after 2 days of culture. Cell viability was performed with Calcein Red-Orange AM (Life Technologies, Carlsbad, CA, USA) as indicated by the supplier after 8 days of culture.

### Direct and indirect cell contact cytotoxicity assays

Cells were used at confluence. Medium was removed at day 0. For direct contact, 800μL of basal solution and 200μL of BSS was directly added. For indirect contact, 200μL of 2% ALGS was placed on the cells during 1 h at 37°C to gellify. Then 800μL of basal solution and 200μL of BSS was added. A condition without biomaterial served as control. The absorbance was estimated by performing MTT assay on day 1 and 4. Cell apoptosis and death was evaluated by Biotium Kit (Annexin V–PI) as indicated by the supplier and with solutions of Annexin V and 7-AAD by FACS analysis after one week of culture. Positive controls were induced by staurosporin.

### Scanning Electron Microscopy examination

30.10^3^ cells were plated and adhered on 2% gelatin coated 24 wells-coverslips for 2 h in 800μL of basal solution. Then, 200μL of BSS was added. Cells cultured in 1 mL of basal medium supplemented with 2% Ethanol served as control and the basal medium was changed every 3 days. After 7 days, coverslips were fixed with 4% PFA / 5% sucrose solution in PIPES for 10 min, rinsed in PIPES and incubated in 2.5% glutaraldehyde in PIPES for 5 min. After rinsing, coverslips were incubated with 0.5% osmium tetroxide for 60 min followed by dehydration in specific grade alcohol, and attached on sample holders and then coated with gold. The samples were examined using Scanning Electron Microscope. Microphotographs were taken with magnifications x400 and x1000.

### Histochemistry

After 7 days in contact with biomaterials, coated coverslips cultures were fixed using 4% PFA / 5% sucrose in PBS for 10 min and were permeabilized using 0.5% Triton X-100 for 5 min at room temperature when needed. To block background staining, cells were treated with 1% BSA/1‰ glycine in PBS at 37°C for 20 minutes. Samples were then incubated with the primary antibody at 4°C overnight or at 37°C for 2 h and with appropriate secondary antibodies at 37°C for 1 h. Cell nuclei were stained using DAPI (Life Technologies Corporation). The antibodies used for immunostaining were: 1/100 monoclonal anti-fibronectin (Sigma-Aldrich) and HFN 7.1 (which was deposited to the DSHB by Klebe, R.J. (DSHB Hybridoma Product HFN 7.1)), 1/150 anti-collagen I (ab292; Abcam), anti-Ki67 (ab15580; Abcam), 1/50 monoclonal anti-vimentin (3CB2 was deposited to the DSHB by De La Rosa, E.J. (DSHB Hybridoma Product 3CB2)), 1/50 monoclonal anti-tubulin. Actin filaments were stained using 1/200 fluorochrome-coupled phalloidin (Life Technologies Corporation). Secondary antibodies were Alexafluor 488-conjugated goat anti-rabbit, Alexafluor 594-conjugated goat anti-mouse, Alexafluor 546-conjugated goat anti-mouse and Alexafluor 488-conjugated goat anti-mouse (Life Technologies Corporation). Samples were observed using a Zeiss microscope equipped with a digital camera.

### Analysis of mRNA expression by RT-qPCR

After 7 days of culture with biomaterials, total RNA was extracted. RNA concentration and purity were evaluated by spectrophotometry (Nanodrop, Thermo Scientific, Wilmington, MA, USA). Total RNA concentration and purity was measured and samples with OD260/280 ratio above 1.8 were used for the study.cDNA was synthesized using the Superscript II kit (Invitrogen) according to the manufacturer’s instructions. For the qRT-PCR reactions, succinate dehydrogenase complex subunit A flavoprotein (SDHA), ubiquitin C (UBC) were used as reference genes on the Bio-Rad CFX manager software. Non-transcribed RNA samples and a water control were used as negative controls. The datas were analyzed based on the comparative 2ΔΔCt method (CFX Manager Software Version 2.1, Bio-Rad Laboratories). For primer sequences, see Table 2.

**Table 2:** Primer sequences used for the study.

Genes	Primers sequences (5’>3’)
*SDHA*	F: AGC AAG CTC TAT GGA GAC CT R: TAA TCG TAC TCA TCA ATC CG
*UBC*	F: GTG GCA CAG CTA GTT CCG T R: CTT CAC GAA GAT CTG CAT TGT CA
*IL1-β*	F: GTC TTC AAC AAG ATA GAA GTC AAG R: AGG TGC TGA TGT ACC AGT T
*MMP9*	F: ACA AGG ACA AGC TCT ACG GCT TCT R: TTT ATC AGG GCA GAA GCC CCA CTT

### Statistical analysis

Two primary cell lines of DPSC and of GSC were used in this study. All culture experiments were at least reproduced twice in triplicate. Statistical analysis was performed using one-way ANOVA (Kruskal Wallis modified Dunns test) and was carried out using Prism GraphPad 5 software (GraphPad Software Inc, La Jolla, CA, USA). Results are presented as mean ± standard deviation (SD). Results were considered significant with a *p* value <0.05. (on graphs *: p<0.05; **: p<0.01; ***: p<0.001; ****: p< 0.0001).

## Results

### Biomaterial characterization

**Fig 1:**
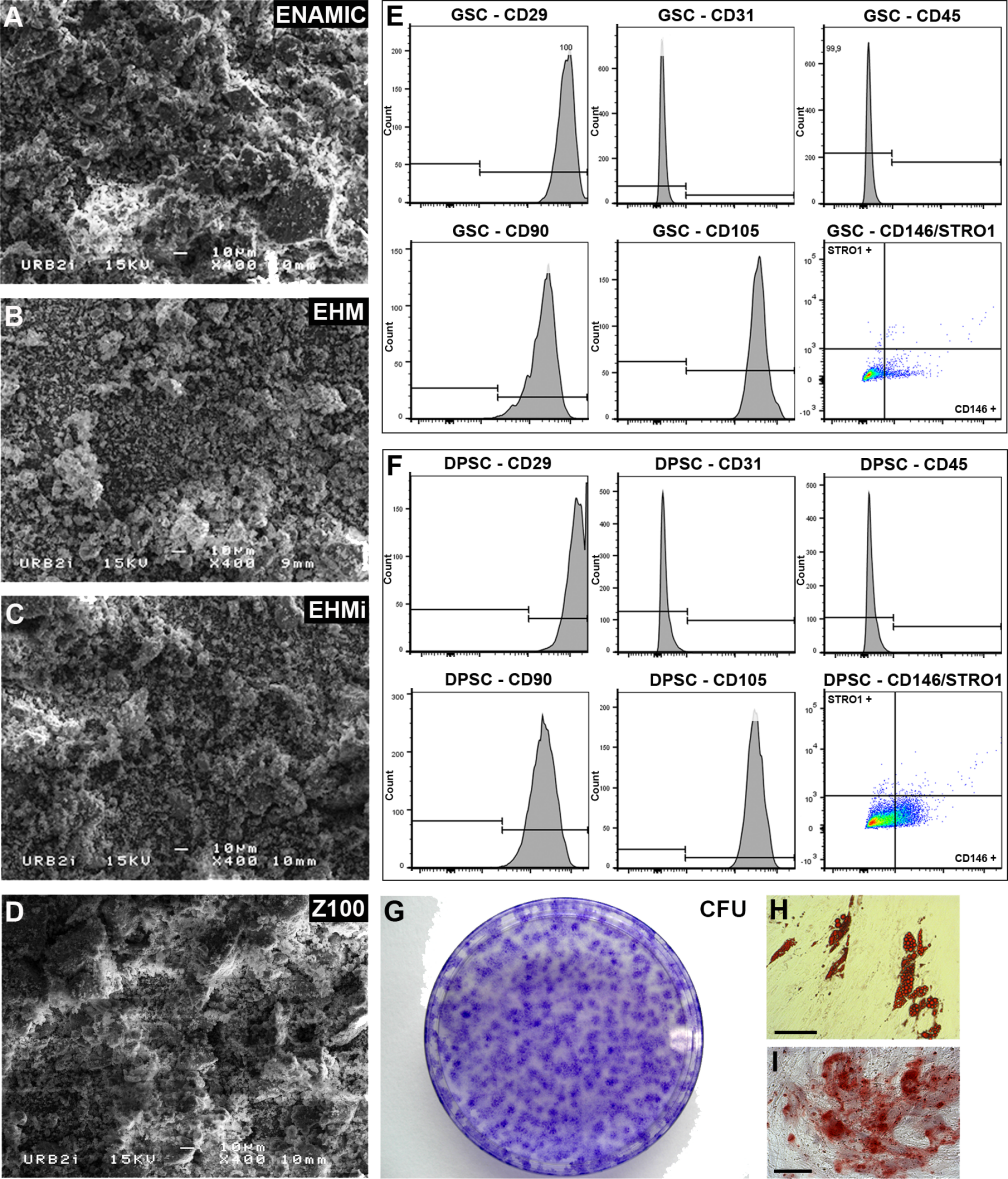
Biomaterials and stem cells characteristics. SEM microphotographs of the powdered materials. Enamic (Fig 1A), EHM (Fig 1B), EHMi (Fig 1C) and Z100 (Fig 1D). FACS analysis of classical mesenchymal stem cells markers (CD29, 90, 105, 146 and STRO1), of hematopoietic cells (CD45) and of endothalium (CD31) for GSC (Fig 1E) and DPSC (Fig 1F). CFU-F assays of the GSC (Fig 1G). Differentiation staining of the GSC after 14 days: Oil Red O staining (Fig 1H) and Alizarin Red S staining (Fig 1I).Scale bar 100μm.

Biomaterials differed in their monomer composition: Bis-GMA and TEGMA for Z100, a mixture of UDMA and TEGMA monomers for Enamic while EHM and EHMi contained only UDMA monomers. Their fillers also varied: zirconia silica for Z100, feldpath ceramics for Enamic and Vita Mark II™ for EHM and EHMi (Table 1). Only EHM did not contain initiators of polymerization. SEM appearance differed little between the materials (Fig 1).

### Stem cell characterization

GSC and DPSC were positive for the mesenchymal stem cells classical markers: CD29, CD90 and CD105. They were negative for the lineage associated markers: CD31 and CD45. Few of them were positives for Stro1 and CD146 markers (Fig 1). GSC cells formed CFU-F by limiting dilution (Fig 1) and were able to differentiate into adipocytes (Fig 1H) and osteoblasts ((Fig 1) after 14 days. DPSC were able to form CFU and mineralized matrix in the same conditions (Data not shown).

### Cytotoxicity

**Fig 2:**
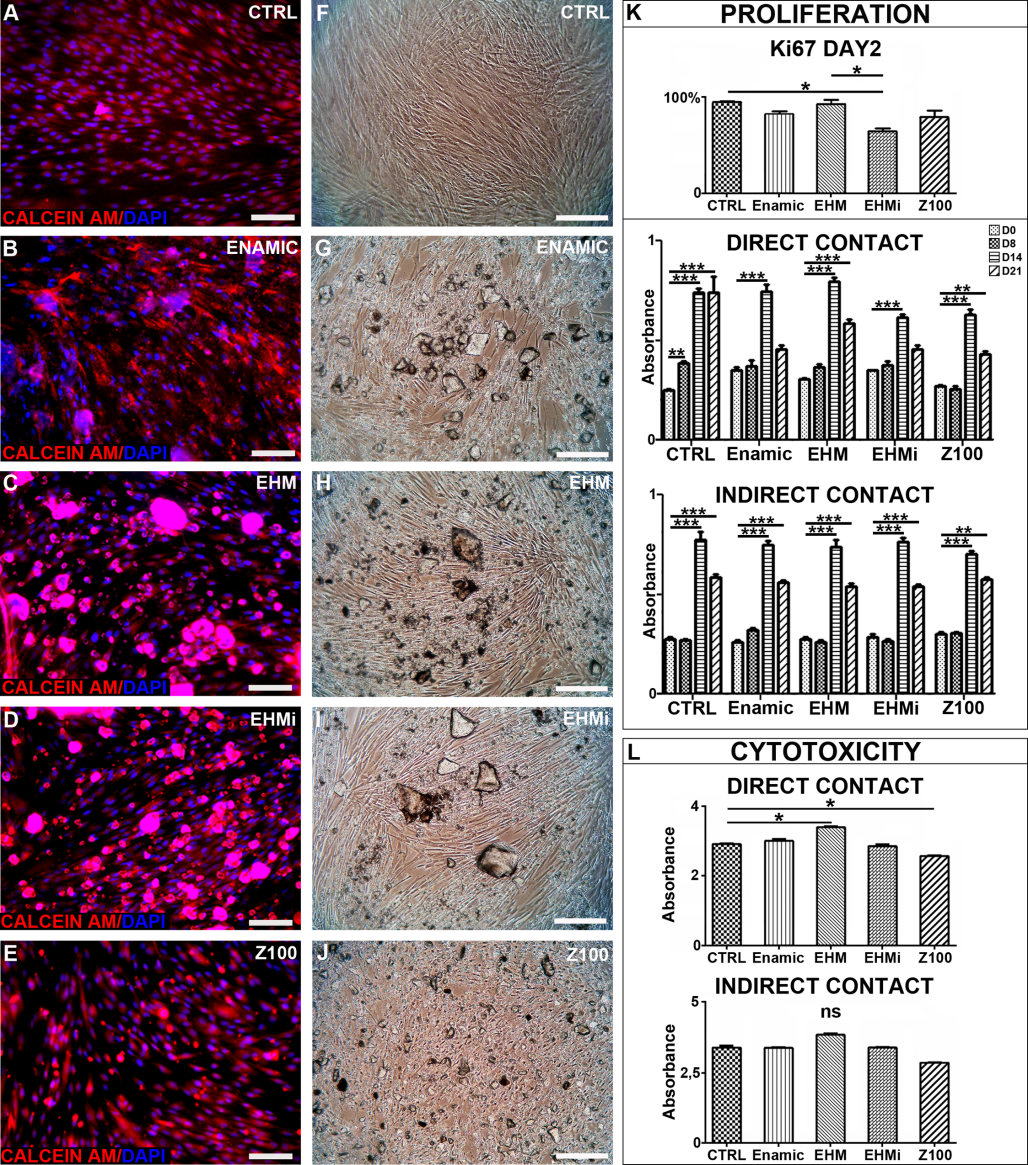
Proliferation and cytoxicity analysis. Calcein AM viability assays for GSC cultured with the tested materials (Fig 2A-E). Cells microscopic morphology in the same conditions (Fig 2F-J). Scale bar 100μm. Figure 2K: Cellular proliferation analysis. Ki67 indexes graph for the 2 first days of culture in direct contact condition; MTT assays in direct contact or indirect contact for 21 days,. MTT cytotoxicity assays in direct and indirect contact conditions (Fig 2L).

Calcein AM fluorescence revealed that most of the cells in contact with biomaterials were alive (Fig 2) like in control condition (Fig 2). After one week, cell cultured with the biomaterials were confluent like controls (Fig 2) and no dead cells were observed. Non-confluent cells were also exposed to the biomaterials and proliferation, monitored (Fig 2). After 2 days of culture, only EHMi Ki-67 index differed from the control culture and was also different from EHM condition. Proliferation analysis revealed that in all conditions cells reached confluency at day 14. In contact with materials, cells number decreased on day 21. To understand if direct contact or diffusion would be involved, we reproduced the same experiment with the biomaterials lying on an agarose gel to avoid direct contact with the cells. In these indirect conditions, the cells reached confluency at day 14 then reached a plateau on day 21 lower than day 14 but significantly higher than initial cell number. In conclusion, the studied materials did not seem to greatly influence cell proliferation. Raw datas can be found in S1 Fig.

To further analyze cytotoxicity, we performed MMT assay on confluent cells exposed to the biomaterials for 96 hours (Fig 2). We noticed a significant decrease of MTT absorbance only for the Z100 condition. In indirect condition, no significant differences were observed. This underlined the requirement of a direct contact in the cytotoxic effect observed under Z100 exposure.

### Apoptosis and cell death

**Fig 3:**
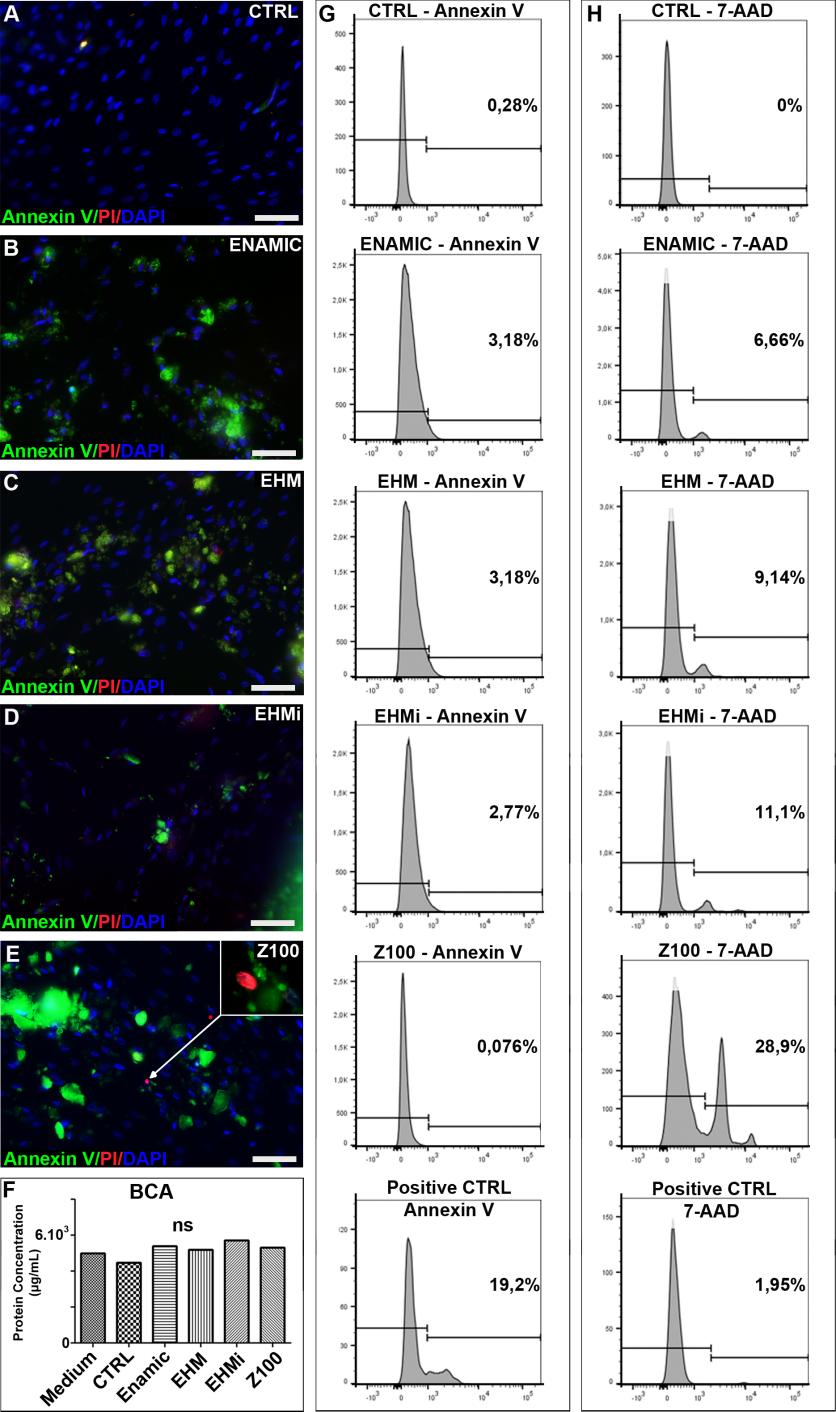
Apoptosis and cell death in contact with the biomaterials. Detection of apoptosis and cell death. Annexin V and propidium iodide fluorescent stainings of the GSC cultured with no materials (Fig 3A), Enamic (Fig 3B), EHM (Fig 3C), EHMi (Fig 3D) and Z100 (Fig 3E). BCA measurement of the protein concentration in the respective culture conditions (Fig 3F). Flow cytometry analysis of the apoptosis marker Annexin V (Fig 3G) and of dead cells 7-AAD (Fig 3H). Scale bar 100μm.

Cells were incubated for a week in contact with biomaterials deposited on cells at 70% of confluence. We assessed apoptosis and cell death through the use of Annexin V and Ethidium bromide (Biotium kit) in immunofluorescence, respectively. Cells showed a fibroblastic morphological appearance unchanged under optical microscope and without cell death evidences in both material-exposed and control conditions. No apoptosis was noticed in culture after contact with biomaterials like in the control, except few dead cells observed in Z100 conditions (Fig 3). To analyse more precisely the cytotoxicity, we performed flow cytometry using AnnexinV and 7-AAD, staurosporin was used as a positive control for apoptosis. Few apoptotic cells were detected (Fig 3). This experiment revealed an induction of cell death in all the materials direct conditions, this was more evident with Z100 (28%) than in Enamic (6.66%), EHMi (11.1%) and EHM (9.14%) (Fig 3).

### Inflammatory response

Cells did not express IL-1β and Matrix metalloproteinase 9 (MMP-9) (data not shown), generally associated with response to inflammation of connective tissue cells. In line with these results, total protein secretion was not modified by biomaterials contact, as measured by BCA assay (Fig 3)

### Cytoskeleton and extra cellular matrix

**Fig 4:**
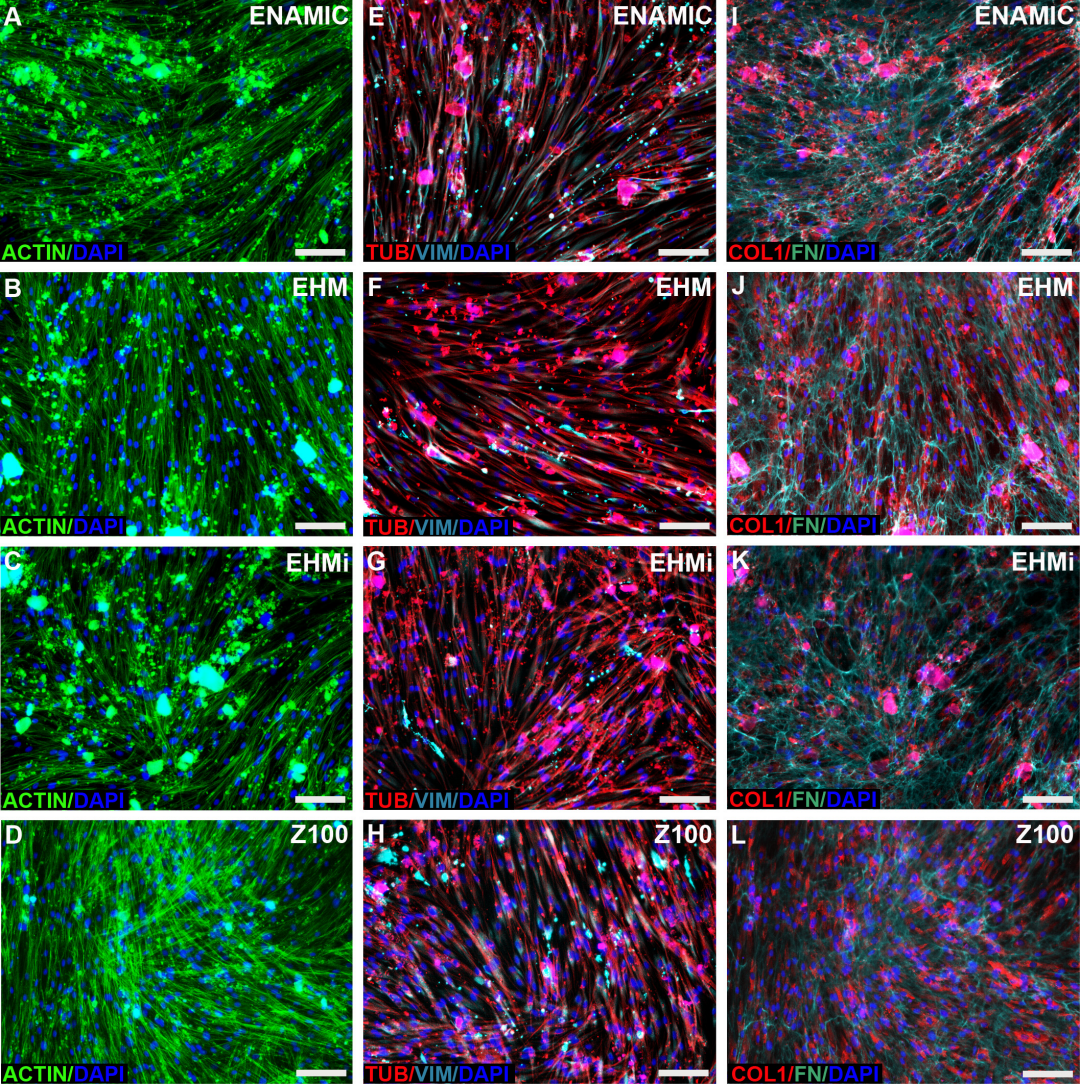
Cytoskeleton and extracellular matrix components analysis. Actin and Dapi fluorescent stainings of GSC cultured with either Enamic (Fig 4A) or EHM (Fig 4B) or EHMi (Fig 4C) or Z100 (Fig 4D). Tubulin, Vimentin and Dapi immunofluorescent stainings of DPSC cultured with either Enamic (Fig 4E) or EHM (Fig 4F) or EHMi (Fig 4G) or Z100 (Fig 4H). ProCollagen 1alpha1, Fibronectin and Dapi immunofluorescent stainings of GSC cultured with either Enamic (Fig 4I) or EHM (Fig 4J) or EHMi (Fig 4K) or Z100 (Fig 4L). Scale bar 100μm.

At day 2 or day 8, the main components of the cellular cytoskeleton vimentin (intermediate filament), tubulin (microtubule) and actin did not change between cells cultured with biomaterials and control (Fig 4). Thus, the assessed biomaterials did not disrupt or disorganize cellular architecture. We finally studied the two main secreted molecules in ECM: type I collagen and fibronectin. Their distribution and expression levels did not appear altered in presence of the RIGCN and classical composite materials (Fig 4).

### SEM Examination

**Fig 5:**
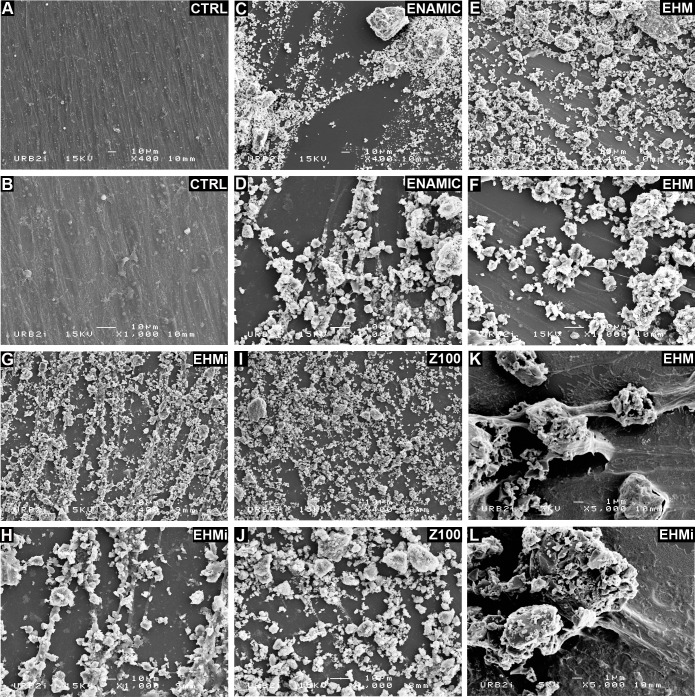
SEM Microphotographs of the studied materials. Z100 (Fig 5I and J), Enamic (Fig 5C and D), EHM (Fig 5E and F), EHMi (Fig 5G and H) and of the cultured cells (Fig 5A and B). Higher SEM magnification of the intereactions between cells and EHM (Fig 5K and L).

SEM examination after 7 days revealed intimate contacts of cells for the materials. But, the adhesion of cells on the material was difficult to assess as the material is in powder. Cell shape was elongated and rather flattened in the control culture (Fig 5). Cells number appeared to decrease, especially for the Z100 (Fig 5). This decrease in cell number may be caused by a physical constraint of the powder which surface condition and numbers of particles were heterogeneous and would diminish the space available for cell growth.

## Discussion

The aim of this study was to evaluate the biocompatibility of three hybrid PICN materials (Enamic™, EHM and EHMi) using the classical resin based composite as a reference and using two distinct oral mesenchymal cell culture models.

We evidenced that RIGCN are less cytotoxic than classical composites (MTT assay and flow cytometry analysis of 7-AAD) and do not induce a decrease in type I collagen secreting cells number. This is of particular interest for future applications. Indeed, blocks of the tested materials are intended to restore damaged teeth. In addition to their mechanical qualities which must be compatible with a sustainable tooth restoration, their appropriate biological properties will be the guarantors of an optimal restauration tolerance. As physical properties of these materials have been previously evaluated [6,16], the present study on their biological effects on oral cells might give some robustness for clinical transfer in dentistry.

It should be kept in mind that these materials will not usually be in direct contact with tissues and cells. First, within the oral environment, the dental restorative biomaterials will interact through biofilms layered on their surface. This biofilm is one of the factors that contribute to surface degradation of composite resins [17] while ceramics retains only a thin biofilm [18]. Despite the contact with gingival tissue is indirect, these biomaterials cytotoxicity is significant to explore because this biofilm behaves like a permeable membrane. Concerning the teeth, a dentin barrier is normally interposed between dental pulp cells and the material, second a layer of bonding agent or cement lays between restoration and dental tissue. However, oral cells and tissues may be challenged in the oral microenvironment. And, materials or more specifically their individual components might reduce their healing ability. In this respect, GSC and DPSC were used here, as candidate cells recruited *in vivo* in case of aggression or inflammation and able to differentiate. More specifically, in conditions where adhesive or dentin barrier is affected, a reparative process is initiated [19]. And, pulp stem cells are actively involved in dentin and pulp tissue regeneration [20]. Consequently, pulp stem cells have been used to test molecules used in restorative dentistry[21]. All these considerations led us to select DPSC and GSC stem cells to evaluate here potential effects of the emerging new CAD-CAM materials.

The components of composite resins have essentially been reported to be toxic when individually applied [22]. Although some of the basic monomers, such as BisGMA or UDMA, are poorly soluble in water, *in vitro* studies have demonstrated that the primary leachable component from composites was UDMA monomer. However, long-time curing, post-curing for 24 hours and removal of the oxygen-inhibited surface layer decrease the cytotoxicity of composite materials[23]. Powders of experimental blocks tested in this study contained UDMA with or without initiator. This monomer is intimately linked to the ceramic network because its polymerization is made under high temperature and high pressure [5]. The resulting blocks are free from surface inhibited layer and their stability, improved [16]. Machining of blocks might raise the temperature and mobilize this monomer. But such an event is unlikely if appropriate cooling of instruments is implemented. In addition, the structure of the material with a single polymer preserves from synergistic cytotoxic effects [24] and easy leaching into the medium, due to the nature of the chemical components and the existing bonds between them[25]. The control material (Z100, 3M) contained BisGMA and TEGDMA. Cell viability at its contact has been shown to be variable, depending on extracting media, with or without ethanol[25]. The results of cytotoxicity testing of the composite powder (direct contact) showed a decrease of living cells which was, albeit slight, statistically significant. In this respect, ethanol-water solution is known to accelerate extraction of composite components[26]. And, short term evaluations have been using high ethanol concentration (75 to 96%) to assess the material extracts [27]. 75% ethanol has a solubility parameter which matches that of BisGMA and promote its release for a longer time window and in higher amount [28]. It should be kept in mind that ethanol extraction media is not physiologically relevant. Thus, our samples were conditioned in a culture medium with only 2% ethanol to improve the contact of the powder with the cells and assessment was made at day 7. These experimental conditions might mimic the oral environment wherein the material is immersed and low ethanol concentration does not interfere with the release of the constituents. Finally, the formulation in powder may be at the origin of the decrease in cell number observed in SEM due to lack of space in cell development[29]. But, *in fine*, biomaterials do not directly inhibit cell proliferation as shown by stable Ki67 expression.

Our study also evidenced that biomaterials did not alter cells cytoskeleton and adversely affect the synthesis of the main extra-cellular matrix components. SEM examinations provided another lightening on cell morphology. Among the variables related to the structure and composition of biomaterials, one of the main parameters is surface topography, as it influences *in vitro* cell behavior in term of cell morphology, proliferation and adhesion[30]. In order to limit surface topography effects, samples were grinded from a polymerized block. Our observations did not check which molecules are involved in cell adhesion to the materials.

Interestingly, all the tested biomaterials did not modify proliferation, extracellular matrix synthesis, morphology or inflammatory response in our experimental conditions. Some studies have reported cytotoxicity of dental composites, which was observed here. In these studies, dental composites components were used before polymerization while here, we used stem cells and polymerized materials [31,32]. Still no adverse effect was reported in dental composite clinical use for the 20 years they have appeared[2]. The classical composite cytotoxicity may be due to monomer microleakage since this latter was not polymerized under high pressure and high temperature. This means a less complete polymerization. Future studies may focus on the polymerization mode influence on cytotoxicity.

The four polymerized and powdered materials studied here showed few differences in our experimental conditions. Only significant cytotoxicity was observed for conventional composites using MTT assay and 7-AAD. Thus, the present study demonstrated that these new CAD—CAM materials would be safe to use in the oral sphere. As their intrinsic mechanical properties have been previously shown to be optimal [5], they would constitute an interesting alternative to ceramics or composite-based blocks for the present restorative dentistry based on CAD–CAM processing.

## Supporting Information

S1 Fig Raw datas and statistical analysis of the proliferations assays. (XLSX)
